# Comparing Castability of Nickel-Chromium, Cobalt-Chromium, and Non-Precious Gold Color Alloys, Using two Different Casting Techniques

**DOI:** 10.30476/DENTJODS.2021.87573.1275

**Published:** 2022-03

**Authors:** Elham Ansarifard, Mitra Farzin, Arghavan Zohour Parlack, Masumeh Taghva, Razieh Zare

**Affiliations:** 1 Dept. of Prosthodontics, School of Dentistry, Shiraz University of Medical Sciences, Shiraz, Iran; 2 General Dentist, School of Dentistry, Shiraz University of Medical Sciences, Shiraz, Iran; 3 Dept. of Oral and Maxillofacial Pathology, School of Dentistry, Shiraz University of Medical Sciences, Shiraz, Iran

**Keywords:** Castability, Chromium Alloys, Non-Precious Gold Color (NPG), Dental Casting Technique; Centrifugation

## Abstract

**Statement of the Problem:**

The castability of nonprecious gold color alloy using torch/ centrifugal and induction/vacuum-pressure casting techniques has not been studied yet.

**Purpose:**

This study was conducted to compare the castability of nickel chromium, cobalt-chromium and nonprecious gold color alloy using torch/centrifugal and induction/ vacuum-pressure casting techniques.

**Materials and Method:**

In this *in vitro* study, a total number of 54 identical acrylic wax meshes were prepared and divided into 6 different groups of 9 each. Group 1: nickel-chromium alloy, which was casted with induction technique. Group 2: nickel-chromium alloy was casted with centrifugal technique. Group 3: cobalt-chromium alloy was casted with induction technique. Group 4: cobalt-chromium alloy was casted with centrifugal technique. Group 5: nonprecious gold color alloy was casted with induction technique. Group 6: nonprecious gold color alloy was casted with centrifugal technique. Then castability of specimens was measured using modiﬁed Whitlock’s method. The results were analyzed using two way ANOVA and post hoc tests.

**Results:**

ANOVA test revealed no statistically significant difference between different alloys with a *p* Value of 0.313. Moreover, it represented no significant differences within the groups regarding alloy types and casting techniques with a *p* Value of 0.511 and 0.682, respectively.

**Conclusion:**

No significant difference was found in the castability value of nickel-chromium, cobalt-chromium, and nonprecious gold color alloys. In addition, the castability value of three alloys tested in this study was not different by using torch/centrifugal or induction/vacuum-pressure casting machines.

## Introduction

Dental casting alloys continue to be used widely in prosthetic restorations. Due to increasing cost of precious metal alloys, use of semiprecious and nonprecious base metal alloys is dominant[ 1 ]. Base metal alloys have the advantages of low specific gravity, greater stiffness, higher hardness, good rigidity even in small thickness and acceptable resistance to tarnish and corrosion relative to noble metal alloys [ 1 ]. Regarding these features, nickel-chromium (Ni-Cr) alloy is used widely in prosthodontic field for crowns and bridges as well as post-core constructions [ 2 ]. Despite its advantages, the use of Ni-Cr alloy has some concerns. One of them is toxic and allergic effects of nickel on human body when exposed to the oral cavity [ 3 ]. One of the alloys that can replace Ni-Cr is cobalt chromium (Co-Cr) alloy. Biocompatibility of Co-Cr alloy has been proven in previous studies [ 4 - 5 ]. Besides, the corrosion of it is lower than Ni-Cr .The casting process of Co-Cr is convenient and it is cost benefit. Co-Cr alloy can be used for metal ceramic restorations; the bond to porcelain is acceptable and distortion during porcelain firing is very low [ 6 - 7 ]. Due to high yield strength of Co-Cr alloy, plastic deformation and porcelain debonding is rare for this alloy [ 8 ]. Nonprecious gold color (NPG) alloy is another base metal alloy, which is introduced in recent years to overcome Ni-Cr alloys limitations. It has surface characteristic of precious type III yellow gold. The manufactures claim that it can be used for full cast crowns, onlays, short span multiple unit bridges, metal substructure for veneer crowns using polymer resins, and post cores [ 9 ]. Khaledi *et al*. [ 10 ] showed that endodontically treated teeth that were restored with NPG posts, had higher fracture resistance than those with Ni-Cr posts. They attributed this property to similarity of elastic modulus of NPG posts and dentin structure, which was confirmed by later studies [ 11 ].

Having an acceptable castability is considered as one factor for accepting an alloy in dentistry. Castability is defined as the ability of a molten metal to completely fill the mold created by the burn out of wax pattern [ 12 - 13 ]. Many factors influence castability of an alloy. For example, type of alloy, wax pattern design, investment ingredients, burnout temperature, casting technique and direction of casting forces [ 14 - 17 ]. Casting of alloys might be done, using two types of casting machines; torch/centrifugal or induction/vacuum-pressure [ 18 ].

There is limited information available regarding castability of NPG alloy and Co-Cr alloy, using different casting techniques. Therefore, the purpose of this study was to compare the castability of Ni-Cr, NPG and Co-Cr alloys, using torch/centrifugal casting machine and induction/vacuum-pressure casting machine. The null hypothesis was that the castability of a dental alloy is not affected by the alloy type and casting machine.

## Materials and Method

**Figure 1:**
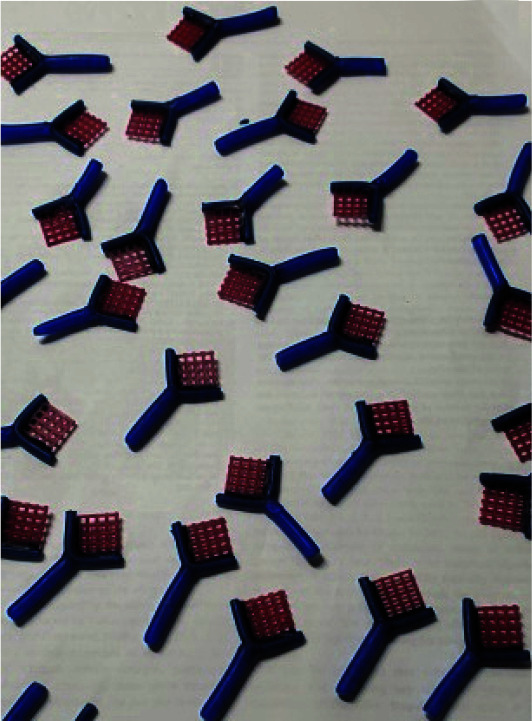
The mesh wax patterns

Castability of the tested alloys in this study follows the standard method of castability, proposed by Whitlock and Hinman in which the ability of an alloy to occupy a mold, which is created by burning out a mesh of nylon, is determined as castability standard test [ 19 ]. A total of 36 Wax patterns were prepared from a wax mesh pattern to design partial denture framework (Dandiran, Iran). The dimensions of each mesh were a square of 11mm* 11mm with 16 square shaped spaces of 2mm* 2mm and filament diameter of 1mm. Two runner bars of 10 gauges (Renfert, Germany) were attached to the two adjacent sides of each mesh. Then a 6-gauge sprue (Renfert, Germany) with the length of 10mm was attached to one corner of the mesh (Figure 1). 

The pattern was attached to the crucible former and sprayed with a surface reducing agent (Lubrofilm, Dentaurum, Germany) and then air dried. Next, the pattern was invested with phosphate bonded investment (Bellasun, Bego) following the manufacturer’s instructions. The wax pattern was left to bench set for 60 minutes. Then, wax burn out was done by keeping the casting ring in the furnace (Magma, Renfert, Germany) and the temperature was raised to 250°C. This temperature was maintained for 1hour. The temperature was raised further to 950°C and maintained for 1hour in order to burn out the wax completely. In this stage, 36 mold cavities were created.

**Figure 2:**
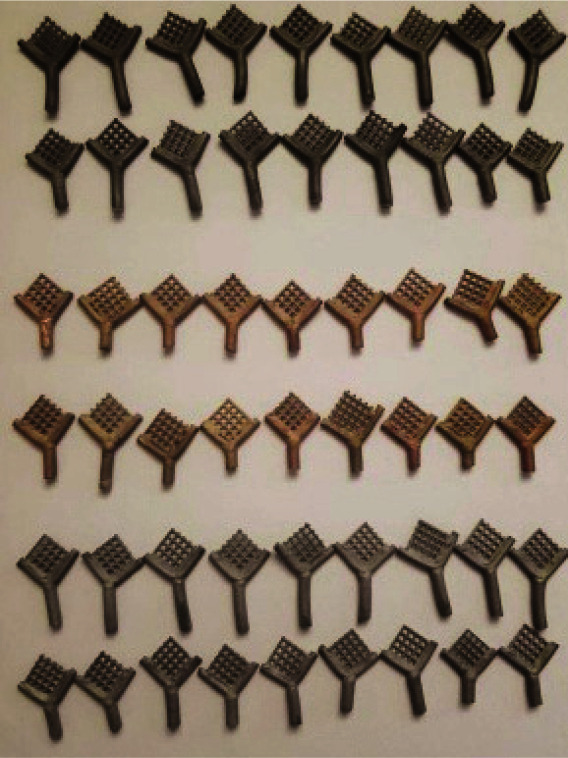
The casted specimens

In the present study, three types of alloy were used for casting including Ni-Cr alloy (4all, Ivoclar Vivadent, Germany), Co-Cr alloy (MESA, Italy), and NPG alloy (AalbaDent, USA). Their chemical composition and properties are described in Table 1. Two casting techniques were performed in the current study including torch/centrifugal casting machine (Centrifi- co; Kerr Manufacturing) and induction/ vacuum-pressure casting machine (Nautilus t, Bego, Germany). Each alloy was casted, using both of these casting techniques. Therefore, six groups of alloys and casting techniques were created presented in Table 2. The casting of the created molds was performed, using each alloy and a casting technique. Casting rings were bench cooled and then divested. Cleaning of the remaining investment was done with aid of ultrasonic and sandblasting with 50 to 70µm alumina particles (Hi-Aluminas, Shofu, Kyoto, Japan) (Figure 2).

**Table 1:** chemical compositions and physical properties of three alloys

Alloy Composition	Classification	Density (g/cm^3^)	Melting Range (Solidus/ Liquids)	Elongation (%)	Vickers Hardness	Color
Nickel-Chromium Ni (61.4%); Cr (25.7%); Mo (11.0%); Si (1.5%); Al, C, Mn (<1.0%)	Base Metal	8.4	1260 - 1350 °C	12	235	White
Cobalt-Chromium Co (64%); Cr (21%); Mo (6%); W (6%); Si, Fe, Mn	Base Metal	8.8	1309-1417 °C	10	286	White
NPG Cu (80.7%); Al (7.8%); Ni (4.3%); Fe, Zn, Mn	Base Metal	7.8	1012-1068 °C	15	140	Yellow-Gold

**Table 2:** Numbers of samples in each group

Metals	Methods	Number
1.Ni-Cr	A:Induction	9
	B:Centrifugal	9
2.Co-Cr	C:Induction	9
	D:Centrifugal	9
3.NPG	E:Induction	9
	F:Centrifugal	9

The Castability value was calculated as described. The wax mesh with 16 square spaces provides 40 segments. The numbers of complete cast segments were counted, and castability value was calculated according to Whitlock suggested equation as follows:

$\mathrm{Castability value}=\frac{\mathrm{Numer of completely cast segmetns}}{40}\times 100$

The effects of alloy type and casting method on product castability were then investigated statistically, using two-way ANOVA. The significance level was set at α=0.05.

## Results

The means and standard deviations of castability value for each group are shown in Table 3. In order to compare the means for each of the study group, Two- way ANOVA test and Tukey HSD tests were used. Two-way ANOVA test revealed no statistically significant interaction between the effects of alloy type and casting technique on castability value (F=1.286, *p*= 0.286). In addition, it represented no significant differences of castability regarding alloy type and casting technique with a *p* Value of 0.108 and 0.516, respectively. The results of pairwise comparisons of castability values by using Tukey HSD are shown in Table 4.

**Table 3:** Mean percentage castability values of three alloys, using two different casting methods

Metal	Method	Mean	Std. Deviation	No.
Ni-Cr	1.Induction	99.16	1.25	9
	2.Centrifugal	98.61	2.2	9
	Total	99.88	1.76	18
Co-Cr	1.Induction	99.72	0.83	9
	2.Centrifugal	100	0	9
	Total	99.86	1.06	18
NPG	1.Induction	99.44	1.66	9
	2.Centrifugal	99.21	1.59	9
	Total	98.88	1.9	18

**Table 4:** Comparing castability values within groups

Groups Compared	*p* Value	Significance
A and B	0.97	No
A and C	0.97	No
A and D	0.86	No
A and E	0.86	No
A and F	0.99	No
B and C	0.65	No
B and D	0.42	No
B and E	0.99	No
B and F	0.86	No
C and D	0.68	No
C and E	0.99	No
C and F	0.97	No
D and E	0.22	No
D and F	0.65	No
E and F	0.65	No

## Discussion

In this study, the castability value of Ni-Cr, Co-Cr and NPG alloys were tested, using both centrifugal and induction casting techniques. The results showed that there was no difference in the castability of these three types of alloys, using each casting technique, and therefore, the null hypothesis was accepted.

Due to increased cost of precious alloys, they are substituted by base metal alloys in dentistry. Ni-Cr is a base metal alloy which is mainly used to construct metal ceramic and cast post restorations [ 2 ]. Some countries replaced Ni-Cr alloy with Co-Cr alloy to restrict Ni allergen effects [ 3 ]. NPG alloy is another alloy which was introduced several years ago, with favorable characteristics [ 9 ].

Whitlock's method was used in the present study to measure castability value of Ni-Cr, Co-Cr, and NPG alloys. The castability value of various alloys and casting techniques was measured using different methods [ 20 ]. However, it was found that Whitlock's method is easy and does not require any special equipment to assess the castability. Furthermore, the size and the shape of each specimen can be easily standardized [ 19 ]. In this method, the number of completely casted segments is divided by total number of segments of the nylon mesh. Since the nylon mesh with the required dimensions was not available, Whitlock’s method was followed with a minor modification, which was the use of acrylic-wax mesh instead of polyester sieve cloth [ 21 ].

The mean castability values (in percentage) of the three alloys in the present study casted by using centrifugal and induction procedures were between 98.88% and 99.88%. The mean castability value of the tested alloys was close to ideal value of castability. Palaskar *et al*. [ 22 ] and Sharma *et al*. [ 23 ] evaluated the effect of recasting of Ni-Cr alloy, using the induction technique. The mean castability value of new alloy in these studies was 99.36% in the first study and 100% in the second study. The castability value of Ni-Cr alloy by induction method in the current study was 99.88%, which was in line with the mentioned studies.

Imran *et al*. [ 24 ] evaluated the effect of reused Co-Cr alloy on its castability value, using torch centrifugal method. Their result showed no significant difference of castability value amongst the tested groups. The mean castability value of new alloy was 100% in this study. These results are in agreement with the present study, where the mean castability value of Co-Cr in centrifugal method was 100%.

Carreiro *et al*. [ 25 ] compared the castability of Ni-Cr and Co-Cr alloys (Remanium 2000) which is mainly used for the framework of metal ceramic restorations and found that the castability of Remanium 2000 was less than the Ni-Cr. This finding is in contrast with present study, which might be due to different composition of tested alloys. The Co-Cr alloy in the present study consisted of Co (64%), Cr (21%), Molybdenium (6%), vanadium (6%), Si, Fe, and Mn. However, Carreiro *et al*. used Co (61%), Cr (25%), Molybdenium (5%), vanadium (1.5%) and Si composition. 

A study by Thompson *et al*. [ 26 ] evaluated the effect of both torch/centrifugal and induction/vacuum-pressure casting machines on the castability of four dental casting alloys (Genesis II, Liberty, Olympia, Jelenko O). Genesis II is a nickel-free cobalt-chromium-molybdenum base metal alloy, and found no difference in the castability of Genesis II, using the two casting techniques, which is compatible with the results of the current study. However, the castability index between Genesis II and Olympia (gold-palladium high noble alloy) was different, which could be related to different alloy ingredients [ 26 ]. 

The difference between the castabilities of alloys might be related to several items, such as composition, density, surface tension, fluidity and permeability of the alloy, casting pressure, casting method, investment composition, the mold temperature, and melting atmosphere [ 27 ]. One of the limitations of this study was that the specimens were in the form of mesh and not the actual restoration. However, the method to measure castability of the actual form of restoration has not been introduced, yet. Further studies should be carried out to investigate the physical properties of Co-Cr and NPG alloys, using different casting techniques. 

## Conclusion

Within the limitations of this study, it can be concluded that no significant difference was found in the castability value of Ni-Cr, Co-Cr, and NPG alloys. Also, the castability value of the three tested alloys in this study was not different when using torch/centrifugal and induction/vacuum-pressure casting machines.

## Acknowledgement

The authors thank the vice-chancellery of Shiraz University of Medical Sciences, for supporting the research (Grant#9098317). This manuscript is presenting the thesis of Dr. Arghavan Zohour Paralak .Also the authors thank Dr. Mehrdad Vossoughi from the Dental Research Development Center, for the statistical analysis, and wish to thank Mr. H. Argasi at the Research Consultation Center (RCC) of Shiraz University of Medical Sciences for his invaluable assistance in editing this manuscript. 

## Conflict of Interest

The authors declare that they have no conflict of interest.

## References

[1] Roach M ( 2007). Base metal alloys used for dental restorations and implants. Dent Clin North Am.

[2] Bahri D, Sadrnezhaad SK, Koosha S, Najmoddin N ( 2020). Shear Bond Strength of Porcelain Veneering to Nickel-Chromium, Chromium-Cobalt, Zirconia and Lithium Disilicate. J Islamic Dent Assoc Iran.

[3] Lee JJ, Song KY, Ahn SG, Choi JY, Seo JM, Park JM (2015). Evaluation of effect of galvanic corrosion between nickel-chromium metal and titanium on ion release and cell toxicity. J Adv Prosthodont.

[4] Kim HR, Kim YK, Son JS, Min BK, Kim KH, Kwon TY ( 2016). Comparison of in vitro biocompatibility of a Co–Cr dental alloy produced by new milling/post-sintering or traditional casting technique. Materials Letters.

[5] Ramírez-Ledesma AL, Roncagliolo P, Álvarez-Pérez MA, Lopez HF, Juárez-Islas JA ( 2020). Corrosion Assessment of an Implantable Dental Co-Cr Alloy in Artificial Saliva and Biocompatibility Behavior. J Mat Engin Perform.

[6] Eliasson A, Arnelund CF, Johansson A ( 2007). A clinical evaluation of cobalt-chromium metal-ceramic fixed partial dentures and crowns: A three- to seven-year retrospective study. J Prosthet Dent.

[7] Barghi N, McKeehan-Whitmer M, Aranda R (1987). Comparison of fracture strength of porcelain-veneered-to-high noble and base metal alloys. J Prosthet Dent.

[8] Al Jabbari YS ( 2014). Physico-mechanical properties and prosthodontic applications of Co-Cr dental alloys: a review of the literature. J Adv Prosthodont.

[9] Aalbadent, Products, VeraBond Ceramic Alloys. https://aalbadent.com/products/c-rown-bridge-alloys-fmc/npg.

[10] Khaledi AAR, Sheykhian S, Khodaei A ( 2015). Evaluation of retention of two different cast post-core systems and fracture resistance of the restored teeth. J Dent.

[11] Gholami F, Kohani P, Aalaei S ( 2017). Effect of nickel-chromium and non-precious gold color alloy cast posts on fracture resistance of endodontically treated teeth. Iran Endod J.

[12] Presswood RG ( 1983). The castability of alloys for small casting. J Prosth Dent.

[13] Guttal SS, Patil NP ( 2007). Effect of sprue design on the castability and internal porosity in pure titanium castings. Quintessence Int.

[14] Gomathi G ( 2018). A comparative analysis of the influence of new and recast alloy combinations on castability, chemical analysis, microstructural properties of Ni-Cr Alloy. IOSR J Dent Med Sci.

[15] Watanabe I, Woldu M, Watanabe K, Okabe T ( 2000). Effect of casting method on castability of titanium and dental alloys. J Mater Sci Mater Med.

[16] Satoh Y, Toyoma H, Ohki K, Kimura K, Shina Y, Mawda K ( 1989). Studies on the castability of Co-Cr alloy for cast plates. Part 1: The effects of sprue attachment, direction to the wax plates and investment direction of refractory models. J Niton Univ Sch Dent.

[17] Satoh Y, Miyata M, Ono F, Ujiie X, Toyoma H, Ohki K, et al ( 1990). Studies on the castability of Co-Cr alloy for cast plates. Part 2: effect of form of sprue attachment to the wax pattern. J Niton Univ Sch Dent.

[18] Shanley JJ, Ancowitz SJ, Fenster RK, Pelleu GB Jr ( 1981). A comparative study of the centrifugal and vacuum-pressure techniques of casting removable partial denture frameworks. J Prosthet Dent.

[19] Hinman RW, Tesk JA, Whitlock RP, Parry EE, Durkowski JS (1985). A technique for characterizing casting behavior of dental alloys. J Dent Res.

[20] Vaillant-Corroy AS, Corne P, De March P, Fleutot S, Cleymand F ( 2015). Influence of recasting on the quality of dental alloys: A systematic review. J Prosthet Dent.

[21] Palaskar J, Nadgir DV, Shah I ( 2010). Effect of recasting of nickel: chromium alloy on its castability. J Indian Prosthodont Soc.

[22] Palaskar J, Nadgir DV, Shah I ( 2010). Effect of recasting of nickel: chromium alloy on its castability. J Indian Prosth Soc.

[23] Sharma A, Rodrigues SJ, Shetty TB, Shenoy VK, Mundathaje M, Saldanha S ( 2016). Evaluation of effect of recasting of nickel-chromium alloy on its castability using different investment materials: An in vitro study. Indian J Dent Res.

[24] Imran M, Raza M, Sartaj Khan M, Hayat Y ( 2017). Effect of Cobalt-Chromium Alloy Re-Use in Dentistry on its Castability Value. J Ayub Med Coll Abbottabad.

[25] Carreiro Ada F, Ribeiro RF, Mattos Mda G, Rodrigues RC ( 2005). Evaluation of the castability of a Co-Cr-Mo-W alloy varying the investing technique. Braz Dent J.

[26] Thompson GA, Luo Q, Hefti A ( 2013). Analysis of four dental alloys following torch/centrifugal and induction/vacuum-pressure casting procedures. J Prosthet Dent.

[27] Martignoni M, Schönenberger A (1990). Precision Fixed Prosthodontics: Clinical and Laboratory Aspects.

